# How Can Data Drive Policy and Practice in Child Welfare? Making the Link in Canada

**DOI:** 10.3390/ijerph14101223

**Published:** 2017-10-14

**Authors:** Barbara Fallon, Joanne Filippelli, Tara Black, Nico Trocmé, Tonino Esposito

**Affiliations:** 1 Factor-Inwentash Faculty of Social Work, University of Toronto, 246 Bloor Street West, Toronto, ON M5S 1V4, Canada; joanne.filippelli@utoronto.ca (J.F.); tara.black@utoronto.ca (T.B.); 2 Centre for Research on Children and Families, McGill University, 3506 University Street, Montreal, QC H3A 2A7, Canada; nico.trocme@mcgill.ca; 3 School of Social Work, University of Montreal, 3150 Jean Brillant, Montreal, QC H3T 1J7, Canada; tonino.esposito@umontreal.ca

**Keywords:** child welfare, university–child welfare agency partnerships, participatory research, administrative data, research capacity

## Abstract

Formal university–child welfare partnerships offer a unique opportunity to begin to fill the gaps in the child welfare knowledge base and link child welfare services to the realities of practice. With resources from a knowledge mobilization grant, a formal partnership was developed between the University of Toronto, clinicians, policy analysts, and researchers from child welfare agencies across Ontario. The key objectives of the grant included: (1) enhancing the capacity of service providers to access and analyze child welfare data to inform service and policy decisions; (2) integrating clinical expertise in service and policy decisions; and (3) developing a joint research agenda addressing high-priority knowledge gaps. This partnership was an opportunity to advance the evidence base with respect to service provision in Ontario and to create a culture of knowledge and evidence that would eventually support more complex research initiatives. Administrative data was analyzed for this partnership through the Ontario Child Abuse and Neglect Data System (OCANDS)—the first child welfare data system in Ontario to track child welfare-involved children and their families. Child welfare agencies identified recurrence as an important priority and agency-driven analyses were subsequently conducted on OCANDS generated recurrence Service Performance Indicators (SPI’s). Using an urgent versus chronic investigative taxonomy for analyses, findings revealed that the majority of cases did not recur within 12 months and cases identified as chronic needs are more likely to return to the attention of child welfare authorities. One of the key outcomes of the partnership — helping agencies to understand their administrative data is described, as are considerations for next steps for future partnerships and research.

## 1. Introduction

The Canadian Incidence Study of Reported Child Abuse and Neglect (CIS) is the only national source of data on child welfare services in Canada [1]. With the exception of the National Incidence Study (NIS) conducted in the United States, the CIS is the only other serial study in North America that collects data regarding both the extent and nature of child maltreatment through the utilization of surveys of child welfare workers [2]. Three cycles of the CIS have been completed to date (CIS-1998, CIS-2003, CIS-2008) [3,4,5]. As part of the CIS cycles, provincial/territorial incidence studies explore the incidence of reported child maltreatment and the characteristics of children and families investigated by provincial/territorial child welfare services. The incidence studies have provided valuable information to guide policy-makers and practitioners on child maltreatment and the child welfare system’s response. Rates and characteristics of investigated maltreatment vary over time and between provinces. In Canada, rates of investigated child maltreatment have nearly doubled from 135,261 maltreatment investigations in 1998 to 235,842 maltreatment-related investigations in 2008, a rate of 39.2 maltreatment-related investigations per 1000 children [4,5].

Despite the important contributions of the incidence studies, the data collected is cross-sectional, designed to provide national and provincial estimates [1]. Gaps remain in our basic understanding of children and their families who are involved in child welfare services over time, including the long-term trajectories of children and the impact of services on children’s development and well-being [6]. These gaps are barriers to making informed practice and policy decisions [1,6]. Decision-making that is responsive to the needs of child welfare-involved children and families is dependent upon quality data, research evidence, and an organizational culture and climate that supports evidence to inform decisions [6,7]. Formal university–child welfare agency partnerships offer a unique opportunity to begin to fill the gaps in the child welfare knowledge base and to link child welfare research with the realities practice [1,6,8].

With resources from the Social Sciences and Humanities Research Council of Canada’s (SSHRC) Connections Grant (2015–2016), a formal partnership was developed between the University of Toronto, clinicians, policy analysts, and researchers from child welfare agencies across Ontario. This partnership was an opportunity to advance the evidence base with respect to service provision in Ontario and to contribute to a culture of knowledge and evidence that will eventually support more complex research initiatives. This article describes the university–child welfare agency partnership within a complex Ontario child welfare landscape. It discusses how timely, field-directed analyses of pressing issues experienced by this sector is critical to informing child welfare practice and policy responses. One of the rich sources of administrative data analyzed as a foundation for this partnership was the Ontario Child Abuse and Neglect Data System (OCANDS)—the first provincial child welfare data system in Ontario to track child welfare-involved children and families longitudinally. As an example of the type of analyses conducted by this partnership, the results of agency-driven analyses of recurrence using a taxonomy developed by university researchers are reported and implications are discussed.

## 2. Ontario Child Welfare: The Data, Practice and Policy Landscape

Child welfare in Canada is a mandatory service [9] that is governed by legislation specific to each of Canada’s 10 provinces and three territories [2,9,10,11]. The collection of maltreatment statistics in Canada varies across provincial and territorial jurisdictions. In Ontario, estimates of the cost for funding the child welfare system are over 1.4 billion dollars a year [12]. There are 47 child welfare organizations in Ontario and each organization is a not-for-profit, independent legal entity, governed by an independent and volunteer community board of directors [12]. Child welfare agencies across Ontario vary in the delivery of their services [9] and in their information technology systems. A common province-wide information system, the Child Protection Information Network (CPIN) has yet to be fully implemented. The full implementation of CPIN across the province has been delayed due to challenges associated with integrating the different and independent information systems used by organizations to document case and financial management [12]. Moreover, provincial information systems have not typically been used to generate the kind of data required to understand service trajectories, outcomes, and the impact of community level risk factors.

Researchers at the Factor-Inwentash Faculty of Social Work at the University of Toronto currently collect, support, and maintain the data of the only two available comprehensive child welfare data sources in the province: the Ontario Incidence Study of Reported Child Abuse and Neglect (OIS 1993, 1998, 2003, 2008 and 2013) [13,14,15,16,17] and the Ontario Child Abuse and Neglect Data System (OCANDS). The OIS and OCANDS are critical to advancing knowledge of child welfare services across the province. The OIS is the only source of provincially aggregated child welfare data that describe the characteristics of children and families identified to the Ontario child welfare system and estimates the rate of reported maltreatment in the province. The OIS-2013 contains clinical and service information for an estimated 125,000 children identified to the Ontario child welfare system. OCANDS is the only system in Ontario to longitudinally track children and their families.

These two data sources offer distinct, yet complementary contributions. The OIS studies have informed several key policy initiatives in the Ontario child welfare sector, including the implementation of differential response models and the creation of specialized intimate partner violence teams. These data have also contributed to the understanding of risk assessments in child protection investigations [18] and the recognition of opportunities for prevention and early intervention with children at risk of maltreatment [19,20,21]. In contrast to the cross-sectional nature of OIS-2013 data, the longitudinal design of OCANDS allows for the assessment of trajectories and outcomes of children and families involved with the child welfare system.

### 2.1. A Promising and Underutilized Source of Provincial Child Welfare Data: OCANDS

To date, OCANDS contains data for over one million families identified to the child protection system since 2007. OCANDS extracts and standardizes administrative data from disparate information systems from 40 of 47 participating Ontario Children’s Aid Societies. OCANDS was created to provide Children’s Aid Societies (CASs) with a mechanism to develop a better understanding of the service provision, to track service outcomes, and to ultimately, improve the quality of services [22]. OCANDS has been identified as a valuable but underutilized resource for the sector. The capacity of OCANDS to describe the experiences of children and families throughout their involvement with partner agencies has been identified as an essential component for growth in the Ontario child welfare sector. The OCANDS corresponds to the child welfare service continuum -initial allegation, investigation, corresponding decision-making tools (e.g., safety assessment, risk assessment, family strengths and needs, risk re-assessment, etc.), as well as child placement information (e.g., type and duration of each placement, etc.). Research-ready datasets can be constructed from these data in response to specific administrative or practice questions. OCANDS is a child-specific, event-level, longitudinal database.

Ontario child welfare is in the early stages of providing publically reported data regarding service provision. The shift towards enhanced accountability, transparency and continued quality improvement were factors that lead to the introduction of publically reported performance indicators in Ontario [23,24]. In 2011, 24 Service Performance Indicators (SPIs) were endorsed by the province as a collection of metrics that would represent the key dimensions of child welfare. These service performance indicators reflected the key measurement constructs articulated in the National Outcomes Matrix (NOM) [25] which focused on safety, permanence and wellbeing domains, in addition to agency management [26]. In 2015, the Ministry of Children and Youth Services began publically reporting five performance indicators aggregated from two-thirds of Ontario child welfare organizations. Three of five Ontario’s publically reported SPIs are generated from OCANDS. OCANDS also has a dynamic web-based interface for individual child welfare agencies which affords them the ability to run their own queries with their data. The production and calculation of SPIs is the key task performed by OCANDS to date.

Researchers in the US have long noted the promise and potential associated with the use of administrative data for the field of child welfare (e.g., [27]). Drake and Jonson-Reid [27] suggest that a key benefit derived from the use of administrative data is the ability of administrative databases to facilitate reciprocal relationships between researchers, practitioners and policy makers. The unique perspective of child welfare practitioners can lead to valuable and innovative research questions [27]. Accountability and linkability to other sources of administrative child welfare data are potential considerations for use [27]. In Ontario, there is currently no readily available infrastructure or framework that would allow for the linking of child welfare data to other data sources in other sectors, including children’s mental health and youth justice.

The use of multiple information systems in the Ontario child welfare system has created significant barriers to access to longitudinal administrative data. With adequate resources for infrastructure, OCANDS could be linked with other provincial (e.g., Census) and specialized databases (e.g., Ontario’s Looking After Children), creating one of the largest and most complete child welfare datasets in North America. The Canadian Foundation for Innovation (CFI) has funded the infrastructure for the OCANDS and the Ontario Association of Children’s Aid Societies (OACAS) and the Ministry of Children and Youth Service (MCYS) has provided contract funding for the calculation of service performance indicators. Adequate resources, infrastructure, and sustainability are among the issues that must be addressed in order to ensure that the potential of OCANDS is realized and longitudinal child welfare data generated is meaningfully analyzed and translated into evidence of characteristics, trends, outcomes and trajectories of child -welfare involved children and families. Without adequate resources and investment, the potential of using administrative data will be largely unrealized.

### 2.2. Recurrence in Context

The use of administrative data can help child welfare agencies develop clinical and community profiles of children who are at greatest risk of placement disruption and long-term foster care, as well as profiles of families at the greatest risk of recidivism. The construction of these datasets permit statistical modelling methods that have yielded promising results in foster care research conducted in the United States [28,29,30,31] and, more recently, in Canada with the first province-wide longitudinal studies being conducted in Quebec [32,33,34]. These landmark Canadian studies have identified age-specific factors associated with the timing of placement, for whom placement, placement changes, and family reunification are most likely to occur in the province [32,33,34]. There are also numerous examples in the extant literature that highlight the promise of using administrative data to explore recurrence (e.g., [35,36,37,38,39]). Studies conducted in Quebec, Canada by Hélie and colleagues [36,40] are also notable examples.

The majority of research that has addressed maltreatment recurrence originates from the US [41]. US studies show wide variations with respect to rates of recurrence and have ranged from approximately 13% to 69% [41]. There are numerous considerations when comparing studies or integrating study findings on recurrence. Notably, the extant literature is different in study methodology, including the definition of recurrence and the follow-up period of study which make comparisons difficult. Risk factors associated with child maltreatment recurrence include family, child, environmental factors [35,42,43]. Factors related to recurrence include the type of maltreatment (e.g., neglect), chronicity (e.g., number of prior reports), younger children, caregiver risk factors (e.g., substance and alcohol misuse) and family and environmental factors (e.g., lack of social supports, socio-economic or financial status of family) [42]. Disparate policy, legislative and practice contexts between Ontario other jurisdictions makes comparability and generalizability of recurrence findings challenging.

Recurrence has been used as an indicator for child safety in various jurisdictions, including, the United States, Australia, UK, Sweden, and Canada [41]. In the US, national child welfare performance measures are authorized through federal legislation [42,44]. In contrast to the recent emergence of public reporting of recurrence as indicators in Ontario, recurrence is considered an established indicator for child welfare system performance and functioning in the US [42,43,44]. It is part of the US Federal Child and Family Services (CFSR) review process and annual reports by the federal government have defined recurrence as a further incident of substantiated or indicated maltreatment within a six-month period [42,43]. Using this six-month definition, US recurrence rates have been shown to vary between states from 1% to 12% [42]. The importance of conducting research that is applicable and generalizable to the Ontario child welfare context to inform practice and policy is warranted.

## 3. Supporting A University–Child Welfare Agency Partnership for Translating Data into Evidence: The SSHRC Connections Grant

The child welfare sector in Ontario has struggled to make progress in working with and utilizing existing data for the day-to-day operations of the agency and for the evaluation of child welfare practice and policy. This is a significant barrier to enhancing accountability and transparency for services provided. Administrative and Census data have the potential to assist researchers and service providers to better understand child welfare services; however, significant resources, research, and analytic training and capacity are needed to utilize these data [1,6,9]. Trocmé and colleagues have noted that the road to evidence-based practice in the field of child welfare is complex and high stakes [1]. There are challenges in the creation, mobilization and integration of evidence to guide child welfare practice and policy [1,6,9]. The paucity of child welfare research in Canada has been attributed to several interrelated factors, including: limited resources and support for research; poor social work research training; minimal research and evaluation capacity within organizations; and, challenges inherent in conducting research with high-risk populations [1]. There is also limited capacity and infrastructure to utilize data to conduct longitudinal research that informs our understanding of children’s long-term service trajectories and outcomes [1,6]. Although there are examples noted in the extant literature of formal university–child welfare partnerships in the United States to forward evidence driven decisions in child welfare (e.g., [8,45]); there are few in a Canadian child welfare context (see [1,6,9] that are notable exceptions).

Formal university–child welfare agency partnerships can provide an opportunity to place the research infrastructure of the university and the expertise of scholars at the service of child welfare agencies [1]. These partnerships can facilitate the building of capacity to co-create and mobilize research in ways that are relevant and accessible [1,6] and help bridge research to practice [45]. By strengthening data utilization capacity and culture within organizations, university-based research projects are, in turn, more likely to succeed. The literature converges on critical elements that contribute to the development and sustainability of successful collaborative community-academic partnerships: respect and trust amongst partners; having a shared vision, goals and/or mission; a relationship where partners work well together; reciprocity between partners; and, mutual benefits for all partners [46].

Using existing sources of data to answer clinically relevant research questions is critical to building child welfare research capacity within agencies and universities, and to advancing the evidence base [9]. Data becomes evidence by aligning questions with relevant information through the right analytic method [7]. Over the course of its one year duration, this initiative built upon a previous SSHRC-funded research partnership that established a collaborative process for knowledge transfer and mobilization between the University of Toronto and the child welfare sector. Child welfare agency partners approached the research team to augment this work and to produce timely, relevant, field-directed analyses of pressing issues in the sector and to inform ongoing reform efforts in Ontario.

The overarching purpose of the SSHRC funded partnership initiative was to continue to support a developing culture of knowledge exchange in the Ontario child welfare sector that will eventually lead to more complex research initiatives. The key objectives of the connections grant included: (1) enhancing the capacity of service providers to access and analyze child welfare data to inform service and policy decisions; (2) integrating clinical expertise in service and policy decisions; and (3) developing a joint research agenda addressing high-priority knowledge gaps. These outcomes were achieved by providing an opportunity for personnel from the child welfare sector to work with the research team to pose questions, engage in a process of refinement, and disseminate the results of their findings. Over time and multiple iterations, the research to practice feedback loop can expand to accommodate more complex and rigorous research.

Through the resources provided by the SSHRC Connections grant, this partnership allowed the field to access OCANDS in order to address relevant policy and practice research questions. The OCANDS longitudinal design allows organizations to access, examine, and compare child protection data over time. The valuable linkages made between researchers and child welfare practitioners through timely and relevant knowledge exchange is vital to inform and direct effective child welfare policy and practice. Building upon previously SSHRC funded research mobilization initiatives; this initiative established a strong collaborative process and foundation for knowledge transfer and mobilization between the researchers at the University of Toronto and the Ontario child welfare sector.

### Conceptual and Methodological Approach of the Partnership Initiative: A Collaborative Data Analysis and Support Approach

This partnership articulated a shared vision and common goals whereby agencies develop their research priorities and academics facilitate their implementation at the individual agency level, through collaboration across agencies and with the participation of our membership organizations. This partnership was driven by the assumption that successful partnerships between sectors must be reciprocal in nature [1]. The posing and refinement of research questions in response to available data and statistical methods, which is typical of investigator-driven research, is combined with a collaborative approach that assumes that the utilization of research knowledge must be rooted in questions that routinely challenge decision-makers in the field [1,9,47,48]. Stakeholders can provide insight that can assist in identifying key issues that inform research [46]. Moreover, once stakeholders develop the necessary analytical skills, they are better positioned to set sustainable research priorities [1]. Responses to research questions and the link between research and practice must be timely and relevant in order to ensure uptake. Thus, addressing the priorities and questions of partner organizations is critical to successful and sustainable partnerships [1,6].

A model conceptualizing the community impacts of collaborative research partnerships in social sciences and health, the Impact Model informed this initiative, as it highlighted the importance of considering the impacts and key functions of this partnership, including: knowledge generation (e.g., activities that lead to increased knowledge); knowledge sharing (e.g., dissemination activities to enhance the utilization of evidence), and research and training (e.g., opportunities for the development of research skills) [49]. Knowledge generation can result from and produce a range of products or outputs through reciprocal influence [49]. This may include the learning acquired by researchers with respect to the types of research questions that reflect the needs and issues relevant to child welfare practitioners and the children and families they serve. This model considers a diverse range of knowledge-sharing mediums, such as websites and peer-reviewed publications [49]. With respect to research education and training, partnerships may often provide training and development opportunities for university-based researchers and community members. Through the collaborative nature of partnerships, this may include engaging community partners in research designs or other aspects of the research. It may also include university-based researchers developing and refining research agendas and designs to align with the needs and realities of community partners.

Although this SSHRC partnership was funded for a short duration of one-year, the goal of the partnership was to move towards success in each of these key areas to build a sound foundation for other university–child welfare agency partnership initiatives. The assumptions that research impacts are multi-directional, multi-dimensional, and evolve over time underpin this model. By strengthening data utilization capacity and culture within child welfare agencies, university-based research projects are, in turn, more likely to succeed. Capacity building and sustainability are intrinsically linked [50].

Recent analyses of Canadian and Ontario incidence studies have provided cross-sectional reporting of the overrepresentation of child welfare service involvement in Ontario by ethno-racial category and have revealed that Aboriginal children or First Nations children are dramatically overrepresented in the Ontario child welfare system [51,52]. The overrepresentation of Aboriginal children in Ontario is reflective of larger trend in the Canadian child welfare system (e.g., [53,54,55,56,57]). The partnership’s collaborative approach is also aligned with the Ownership, Control, Access and Possession (OCAP) principles [58] and the Tri-Council Policy Statement [59]. Research using OCAP principles and TCPS2 must be guided by the questions generated by Aboriginal communities.

## 4. An Example of Child Welfare Agency-Driven OCANDS Analyses: Recurrence by Urgent Protection and Chronic Need

Within the context of this university–child welfare agency partnership, many research questions emerged. Child welfare agencies identified recurrence as an important priority and agency-driven analyses were subsequently conducted on OCANDS generated recurrence Service Performance Indicators (SPI’s). Recurrence was noted as a serious concern requiring analyses in order to promote the safety and well-being of the children it serves. The publically reported SPIs revealed considerable variation between agencies. Agency members of the partnership wanted to better understand the types of investigations and closures that recur. This partnership represented a unique opportunity to understand the variation between agencies through detailed analyses.

At the request of each participating agency, fact sheets were prepared as a tool to help communicate the findings of child welfare agency-driven analyses to various stakeholders in their respective agencies. The fact sheets were jointly authored by child welfare agency personnel and the research team. With the consent of the participating agencies, information sheets were also prepared with aggregated SPI data. The information sheets developed were made publically available through an open access website, the Child Welfare Research Portal (http://cwrp.ca/). The dissemination of knowledge generated by the partnership at the agency-level and in the broader public domain are indicators of positive impacts of this partnership.

Investigative trends have been emerging in Canada that suggest a shift from urgent protection concerns to an increased focus on the consequences of family dysfunction on child development and well-being [60]. The number of investigations in Ontario has dramatically increased from approximately 47,000 in 1993 to 125,000 in 2013 [16]. It has been hypothesized that the increase in investigations may be partly due to a shift in investigative practices, a greater understanding of the adverse consequences of maltreatment, and the types of investigated maltreatment [16,18,60]. In addition, as a proportion of all investigations, urgent protection cases have decreased across CIS cycles of the [60].

Understanding the profile of children and families and distinct service needs through the urgent chronic taxonomy was proposed by Trocmé and colleagues [60] and was a construct identified as one that the partnership wanted to explore, in particular whether it could be used to understand recurrence. This framework categorizes investigations as either urgent protection, where a child’s acute safety is deemed to be at risk or, chronic need, where the child’s development or well-being are at risk due to family dysfunction [60]. For the purpose of this analyses, investigations were classified as urgent protection if: (a) there was a child in the family younger than four and the investigation was for neglect or physical abuse; (b) the primary concern was sexual abuse; or (c) a child had sustained physical harm [60]. Investigations deemed as fitting the chronic need classification focused on investigations relating to risk of ongoing family challenges impacting child development and well-being (e.g., caregiver capacity) and there was no acute threat to safety or forensic considerations. The data elements needed to construct this taxonomy were available (e.g., child age, closing and recurrence dates, alleged concerns, and physical harm) in OCANDS to map and harmonize for the purpose of these analyses.

Investigating child welfare workers can close a case following the initial investigation or the case can remain open and be transferred for further or ongoing child welfare services. Service Performance Indicator (SPI) 4 measures recurrence of verified investigation in the 12-month period after a case is closed at the investigation stage. Service Performance Indicator (SPI) 5 measures recurrence of verified investigation in the 12-month period after a case is closed from the ongoing child welfare services stage. Recurrence refers to families coming back into contact with the child welfare system after file closure. Verification refers to whether it is more probable than not that the originally alleged or new child protection concerns, which includes risk of harm, have occurred or currently exist [61]. For the partnership, SPI 4 and SPI 5 cases were further disaggregated by either urgent protection or chronic need cases. Notably, the rate of recurrence is not equivalent to the rate of re-victimization. A verification decision can mean that the family requires service, or the child was in fact victimized. The verification of risk factors does not necessarily mean that a maltreatment incident occurred.

Table 1 and Table 2 show the recurrence rates for cases classified as either urgent or chronic at both the index and recurrence intervention stages. The 2013/2014 fiscal year is used as an example. The number of cases initially categorized as chronic following investigation was approximately three times higher at 11,936 (76% of all cases) than those that were classified as urgent. Table 1 shows that of the 3759 cases classified as urgent protection at the time of case closure at the investigation stage, 4% recurred as urgent protection cases; whereas, 7% came back to the attention of child welfare authorities as chronic need. Chronic protection cases recurred as urgent at a rate of 2%; whereas, 11% recurred as chronic need. Urgent cases had an overall recurrence of 11%; whereas, chronic cases had an overall recurrence of 13%. The majority of cases did not recur after 12-months (88%).

Table 2 provides data related to cases that were closed following on-going service provision. For SPI 5, the number of cases classified at initially as chronic at closing was almost five times higher at 4522 when compared to cases initially classified as urgent at closing 917 at ongoing services. Of the 917 cases classified as urgent when they were closed, 7% recurred as urgent protection; whereas, 11% recurred as chronic need. Of the 4552 cases classified as chronic at closing, 3% recurred as urgent; whereas, a much larger proportion, 14% recurred as chronic need.

Overall, the majority of cases (83%) did not recur within 12 months. Cases were more likely to return as chronic need cases. Whether exploring recurrence after case closure at investigation or at ongoing child welfare services, the greatest number of cases that do recur are chronic need cases returning as chronic need.

## 5. Discussion

### 5.1. Next Steps: Aligning Services to Identified Needs

Despite decades of significant policy changes informed by cross-sectional studies, surprisingly little is known about the services provided to children and their impact on children’s service outcomes and trajectories in Ontario, Canada. There is a wealth of administrative data that could help to better understand child welfare services and their impact; however, most child welfare agencies do not have the tools or training to utilize these data effectively [1]. The partnership was developed to support and facilitate Ontario child welfare organizations’ capacity to use administrative data. It was also the result of a University-based team of researchers recognizing their research needed to be rooted in questions and issues that were at the forefront of policy and practice.

Significant resources are required to support the promotion of evidence-based practice [1]. The six child welfare agencies contributed in-kind donations that comprised two-thirds of the overall budget for this federal grant. The commitment of significant resources by the partners to the initiative was viewed as an important indicator of the value placed on this formative work. The partnership is viewed as a first step of a much larger and needed joint effort towards aligning services to needs by producing relevant field-directed analyses of pressing issues to inform ongoing child welfare reform in Ontario. In the interim, as illustrated by the recurrence example, there are several benefits and challenges this partnership identified (see Table 3).

### 5.2. Enhancing the Child Welfare Sector’s Access and Capacity to Utilize Reliable and Valid Data

This partnership was designed to increase access to and the use of administrative data to assist agencies to better understand service trajectories and outcomes for children, youth, and families served; thereby, informing the development of responsive services and policies. There are innovative examples of formal university–child welfare sector partnerships in other jurisdictions, including the California Child Welfare Indicators Project (CCWIP) between the University of California at Berkeley and the California Department of Social Services [8] and the Center for State Child Welfare Data at Chapin Hall at the University of Chicago [62]. As evidenced by these collaborations, infrastructure and resources are needed to generate reliable and valid data and to also transform the data into evidence [8]. Continuous quality improvement (CQI) has been used as framework for improving child welfare decision-making [8,62]. It is an expectation of the US federal government that child welfare uses CQI [8]. Both the CCWIP and the Center for State Child Welfare Data at Chapin Hall work with various US states to generate and utilize CQI measures [8]. Variations with respect to CQI models implemented in numerous states [63].

In 2006, significant policy changes were introduced in Ontario that were intended to include the reporting of agencies quality assurance activities within an accountability framework [64]; however, a comprehensive, consistent, province-wide CQI framework has yet to be implemented. Following this initiative, service partners initiated more in-depth file reviews of cases that recurred to explore opportunities for improvement. It is hoped that future university–child welfare initiatives will integrate continuous quality improvement frameworks in efforts. Continuous quality improvement frameworks and initiatives in the US have demonstrated how CQI can also narrow the gap between research and practice [8]. The work of Wulzcyn and colleagues [62] outlines key components of an infrastructure that supports CQI, including data collection and analytic capacity, agency leadership and culture, CQI knowledge and capacity building throughout the organization, supportive agency structure and functions. The success of this Ontario initiative is being used to demonstrate the inherent value of a University-agency partnership to the broader sector. Both the outcomes (an increased understanding of the chronic nature of the issues facing families and its relationship to recurrence) and the process (working closely with agency partners to understand their organizations and practice demands) have been important building blocks in the quest to maximize the use of administrative data through strong research partnerships.

### 5.3. The Development of A Joint Research Agenda: Next Steps for Understanding Recurrence and Identifying High-Priority Knowledge Gaps

Clinicians, administrators, and supervisors of child welfare authorities are in the best position to pose questions of the OCANDS that are relevant to child welfare practice and policy. The partnership identified the examination of service recurrence as a priority. The goal of the child welfare system is to prevent maltreatment; thus, recurrence may mean that the system has not fulfilled its mandate that includes ensuring the child’s safety [35]. Recurrence is also a publically reported SPI in Ontario and it is considered a proxy for service effectiveness; however, there is much that is not known. Examining what a recurrence indicator truly means has been deemed essential [35]. Helié and colleagues noted numerous unanswered questions with respect to recurrence, including whether recurrence can be translated into inadequate child welfare intervention, whether eliminating all recurrence should be the goal, or if recurrence is inevitable for particularly serious situations and issues [35].

The application of an emerging understanding of how child welfare services should respond to the needs of families through the urgent-chronic taxonomy has helped a broader range of Ontario child welfare stakeholders understand that the majority of cases recur because of a chronic, ongoing family issue. Further analyses are needed to assess a broader definition of recurrence than 12-months of case closure. Although cases with chronic concerns are more likely to recur, this definition and its 12-month time frame may still underestimate the number of families and children with more chronic needs.

Provincial comparisons reveal variation in child welfare practice responses in Canada; more specifically, Ontario has the highest rates of maltreatment-related investigations in Canada with 54.05 per 1000 children; whereas, Quebec has the lowest with 13.19 per 1000 children [65]. These variations appear to be influenced by many factors including differences in socio-demographic characteristics, screening and investigation procedures, and clinical practices [65]. In Ontario, SPI analyses revealed variations in service recurrence rates across child welfare agencies ranging from 9% to 32% [22]. Ontario’s 47 child welfare agencies serve diverse populations [22]. Socio-demographic risk factors in the populations served go beyond the mandate of the child welfare agencies [22]. Ontario recurrence data is reflective of the multifaceted relationship between socio-economic hardship and child maltreatment. Socio-demographic factors of the population of the agency’s community were associated with recurrence. This is in keeping with extant literature that has noted associations between socio-economic status and recurrence [42].

Utilizing 2006 Census data, the rate of recurrence for investigations closed at investigation (SPI-4) by agency was significantly associated with socio-economic factors of the agencies catchment area [22]. Higher recurrence and investigation rates were associated with agencies serving a higher proportion of individuals with lower income, a greater proportion of the Aboriginal population, and a greater proportion of lone parent families [22]. An information sheet highlighting contextual considerations for the variations noted in recurrence rates across child welfare organizations in the province was subsequently prepared [22]. Partners identified the need for further examination of risk factors for recurrence and refinement of the analyses using the urgent-chronic classification. Given the overrepresentation of Aboriginal children involved with the child welfare system in a Canadian context, further research exploring recurrence by ethnic identity and Aboriginal status is warranted and identified by the partnership as important next steps.

The findings of SPI analyses that cases classified as chronic tend to have higher recurrence rates than those that are classified as urgent highlight the complex and potentially unmet needs of children and families served by the child welfare system. It is important to continue to monitor recurrence over time. The child welfare system may be experiencing challenges in utilizing traditional service models that emphasizes physical safety, as opposed to child development. For instance, re-reports may highlight the child welfare system’s challenge in addressing or resolving the complex circumstances that resulted in the report [37]. Complex and chronic issues experienced by families may be more difficult for the child welfare system to address in isolation, without accessible and available social services.

### 5.4. Building Capacity in the Field through Training and Mentoring

This partnership represents an important step to develop agency capacity towards sustainability, and provide opportunities for greater integration of research among agencies. Similar to US university–child welfare sector partnerships (e.g., [8]), future child welfare partnerships in Ontario must focus on building capacity for data collection and analytic capacity. More specifically, training and mentoring of students and agency staff will be critical. By strengthening data utilization capacity and culture within these organizations, university-based research projects are, in turn, more likely to succeed.

## 6. Conclusions

Community resources and infrastructure are critical to the child welfare system’s ability and capacity to meet the needs of the children and families that are served. In order to fully implement participatory research particularly with First Nations partners there is a need to address infrastructure requirements for both the agency and university sectors. These findings highlight the challenges of service provision and the importance of examining children’s trajectories and child welfare service patterns over time. The need for conducting research in the Ontario child welfare context is underscored by the varied state of the recurrence literature in terms of both methodology, findings and context [35]. The majority of recurrence literature originates from the United States. Further research is needed to examine factors associated with recurrence and to link recurrence to outcomes for children and families served in an Ontario child welfare practice and policy context. The reciprocal nature of this partnership underscored the importance of university–child welfare partnerships to pursuing research that is agency-driven, timely and relevant. Future formal partnerships are an opportunity to help to generate, extend, enhance and sustain the research and the knowledge that is so critical to developing responsive and effective child welfare practice and policies in Canada. The data provided by OCANDS has highlighted the interdependence of researchers, practitioners and policy-makers. If OCANDS becomes an established resource for the field, further agency-driven research that addresses timely and relevant questions will be possible. This knowledge can assist in strategically targeting services to children and families who have the greatest needs.

## Figures and Tables

**Table 1:** 12-Month service recurrence of child protection concerns after a closed investigation (SPI-4) by urgent or chronic classification for 2013/14 (6 agencies) (As reported by Fallon and colleagues [61]).

Investigation Type	Urgent Index		Chronic Index		Total	
	#	%	#	%	#	%
Urgent recurrence	159	4	227	2		
Chronic recurrence	251	7	1315	11		
Total recurrence **	410	11	1542	13	1952	12
No Recurrence	3349	89	10,394	87	13,743	88
Total Cases Closed *	3759	24	11,936	76	15,695	100

* All cases closed at investigation during the fiscal year (both verified and not-verified). ** All cases closed at investigation during the fiscal year that were re-opened within 12 months of case closure where the allegations of child welfare concern were verified. #: cases.

**Table 2:** 12-Month service recurrence of child protection concerns after closed ongoing protection services (SPI-5) by urgent or chronic classification for 2013/14 (6 agencies) (As reported by Fallon and colleagues [61]).

Investigation Type	Urgent Index		Chronic Index		Total	
	#	%	#	%	#	%
Urgent recurrence	63	7	120	3		
Chronic recurrence	98	11	641	14		
Total recurrence **	161	4	761	17	922	17
No Recurrence	756	82	3761	83	4517	83
Total Cases Closed *	917	17	4522	83	5439	100

* All cases closed at on-going services during the fiscal year (both verified and not-verified. ** All cases closed at on-going services during the fiscal year that were re-opened within 12 months of case closure where the allegations of child welfare concern were verified. #: cases.

**Table 3:** Challenges and benefits of a university–child welfare sector partnership in Ontario.

Benefits	Challenges
Increased access and capacity to use administrative data.	Resources for sustainability of partnerships.
Data access included more people than those whose job responsibility involves the production of data helping to build capacity.	Resources needed for long-term sustained partnerships include stable financial funding and commitment from agencies (e.g., for training and mentoring agency staff).
Development of a joint research agenda.	Resources and infrastructure needed for data systems that facilitate partnerships.
By demonstrating the utility of longitudinal data researchers can help to support a culture of research and the importance of feedback loops.	The creation, maintenance and sustainability of a province-wide, child welfare longitudinal data system requires secured, long-term funding.
Increased utility and applicability.	Integrating CQI frameworks and process without consistent and comprehensive province-wide CQI frameworks and systems.
New constructs such as the urgent-chronic taxonomy can be tested to assess its real world applicability.	
Addressing pressing field concerns as research priorities strengthens all facets of data collection and application.	

## References

[1] Trocmé N., Roy C., Esposito T. (2016). Building research capacity in child welfare in Canada. Child Adolesc. Psychiatry Ment. Health.

[2] Fallon B., Trocmé N., Fluke J., MacLaurin B., Tonmyr L., Yuan Y.-Y., Kamerman S.B., Phipps S., Ben-Arieh A. (2010). Understanding child maltreatment systems: A foundation for child welfare policy. From Child Welfare to Child Well-Being.

[3] Trocmé N., Fallon B., MacLaurin B., Daciuk J., Felstiner C., Black T., Tonmyr L., Blackstock C., Barter K., Turcotte D. (2005). Canadian Incidence Study of Reported Child Abuse and Neglect 2003 (CIS-2003): Major Findings.

[4] Trocmé N., Fallon B., MacLaurin B., Sinha V., Black T., Fast E., Hélie S. (2010). Chapter 1: Study and objectives and scope. Canadian Incidence Study of Reported Child Abuse and Neglect 2008.

[5] Trocmé N., MacLaurin B., Fallon B., Daciuk J., Billingsley D., Tourigny M., Mayer M., Wright J., Barter K., Burford G. (2001). Canadian Incidence Study of Reported Child Abuse and Neglect 1998 (CIS-1998): Final Report.

[6] Esposito T., Trocmé N., Chabot M., Coughlin L., Gaumont C., Delaye A. (2016). Better understand to better serve: A province-wide knowledge mobilization initiative in child protection. Child Indic. Res..

[7] Lery B., Haight J.M., Alpert L. (2016). Four principles of big data practice for effective child welfare decision making. J. Public Child Welf..

[8] Lery B., Wiegmann W., Berrick J.D. (2015). Building an evidence-driven child welfare workforce: A university–agency partnership. J. Soc. Work Educ..

[9] Fallon B., Trocmé N., Wert M.V., Budau K., Ballantyne M., Lwin K. (2015). Increasing research capacity in ontario child welfare organizations: A unique university–child welfare agency partnership. J. Soc. Work Educ..

[10] Courtney M., Flynn R.J., Beaupré J. (2013). Overview of out of home care in the USA and Canada. Psychosoc. Interv..

[11] Mulcahy M., Trocme N. (2010). Children and Youth in Out-of-Home Care in Canada.

[12] Office of the Auditor General of Ontario (2015). Chapter 3: Reports on value-for-money audits, Section 3.02 child protection services—Children’s aid societies. Office of the Auditor General of Ontario Annual Report.

[13] Trocmé N., McPhee D., Tam K.K., Hay T. (1994). Ontario Incidence Study of Reported Child Abuse or Neglect 1993 (OIS-1993).

[14] Trocmé N., Fallon B., MacLaurin B., Daciuk J., Bartholomew S., Ortiz J., Thompson J., Helfrich W., Billingsley D. (2002). 1998 Ontario Incidence Study of Reported Child Abuse and Negect (OIS-1998).

[15] Fallon B., Trocmé N., MacLaurin B., Sinha V., Black T., Felstiner C., Schumaker K., van Wert M., Herbert A., Petrowski N. (2010). Ontario Incidence Study of Reported Child Abuse and Neglect 2008 (OIS-2008).

[16] Fallon B., van Wert M., Trocmé N., MacLaurin B., Sinha V., Lefebvre R., Allan K., Black T. (2015). Ontario Incidence Study of Reported Child Abuse and Neglect 2013 (OIS-2013).

[17] Fallon B., Trocmé N., MacLaurin B., Knoke D., Black T., Daciuk J., Felstiner C. (2005). Ontario Incidence Study of Reported Child Abuse and Neglect 2003 (OIS-2003): Major Findings Report.

[18] Fallon B., Trocmé N., MacLaurin B., Sinha V., Black T. (2011). Untangling risk of maltreatment from events of maltreatment: An Analysis of the 2008 Canadian Incidence Study of Reported Child Abuse and Neglect (CIS-2008). Int. J. Ment. Health Addict..

[19] Fallon B., Ma J., Allan K., Trocmé N., Jud A. (2013). Child maltreatment-related investigations involving infants: Opportunities for resilience?. Int. J. Child Youth Resielience.

[20] Fallon B., Ma J., Allan K., Trocmé N., Jud A. (2013). Opportunities for prevention and intervention with young children: Lessons from the Canadian incidence study of reported child abuse and neglect. Child Adolesc. Psychiatry Ment. Health.

[21] Filippelli J., Fallon B., Trocmé N., Fuller-Thomson E., Black T. (2017). Infants and the decision to provide ongoing child welfare services. Child Adolesc. Psychiatry Ment. Health.

[22] Fallon B., Filippelli J., Black T., King B., Ekins A., Moody B. (2016). Service recurrence performance indicators in Ontario’s children’s aid societies: Contextual considerations. OACAS J..

[23] Government of Ontario Ministry of Children and Youth Services (2015). Growing. Together. Ministry of Children and Youth Services 2013–2018 Strategic Plan.

[24] Commission to Promote Sustainable Child Welfare (2012). A New Approach to Accountability & System Management: Report and Recommendations.

[25] Trocmé N., Esposito T., Mulachy M., Coughlin L., Fallon B., MacLaurin B., Shlonsky A. (2011). National child welfare outcomes indicator matrix (NOM). Child Welfare: Connecting Research, Policy, and Practice, Second Edition.

[26] Commission to Promote Sustainable Child Welfare (2012). Realizing A Sustainable Child Welfare System in Ontario.

[27] Drake B., Jonson-Reid M. (1999). Some Thoughts on the increasing use of administrative data in child maltreatment research. Child Maltreat..

[28] Wulczyn F., Hislop K.B. (2007). Foster Care Dynamics 2000-2005.

[29] Foster E.M., Hillemeier M.M., Bai Y. (2011). Explaining the disparity in placement instability among African-American and white children in child welfare: A Blinder–Oaxaca decomposition. Child. Youth Serv. Rev..

[30] Zinn A., DeCoursey J., George R., Courtney M. (2006). A Study of Placement Stability in Illinois.

[31] Drake B., Lee S.M., Jonson-Reid M. (2009). Race and child maltreatment reporting: Are Blacks overrepresented?. Child. Youth Serv. Rev..

[32] Esposito T., Trocmé N., Chabot M., Collin-Vézina D., Shlonsky A., Sinha V. (2014). Family reunification for placed children in Québec, Canada: A longitudinal study. Child. Youth Serv. Rev..

[33] Esposito T., Trocmé N., Chabot M., Shlonsky A., Collin-Vézina D., Sinha V. (2013). Placement of children in out-of-home care in Québec, Canada: When and for whom initial out-of-home placement is most likely to occur. Child. Youth Serv. Rev..

[34] Esposito T., Trocmé N., Chabot M., Collin-Vézina D., Shlonsky A., Sinha V. (2014). The stability of child protection placements in Québec, Canada. Child. Youth Serv. Rev..

[35] Hélie S., Bouchard C. (2010). Recurrent reporting of child maltreatment: State of knowledge and avenues for research. Child. Youth Serv. Rev..

[36] Hélie S., Laurier C., Pineau-Villeneuve C., Royer M.-N. (2013). A developmental approach to the risk of a first recurrence in child protective services. Child Abuse Negl..

[37] Putnam-Hornstein E., Simon J.D., Eastman A.L., Magruder J. (2015). Risk of re-reporting among infants who remain at home following alleged maltreatment. Child Maltreat..

[38] Drake B., Jonson-Reid M., Sapokaite L. (2006). Rereporting of child maltreatment: Does participation in other public sector services moderate the likelihood of a second maltreatment report?. Child Abuse Negl..

[39] Fluke J.D. (2008). Child protective services rereporting and recurrence—Context and considerations regarding research. Child Abuse Negl..

[40] Hélie S., Poirier M.-A., Turcotte D. (2014). Risk of maltreatment recurrence after exiting substitute care: Impact of placement characteristics. Child. Youth Serv. Rev..

[41] Bae H., Kindler H. (2017). Child maltreatment re-notifications in Germany: Analysis of local case files. Child. Youth Serv. Rev..

[42] White O.G., Hindley N., Jones D.P. (2015). Risk factors for child maltreatment recurrence: An updated systematic review. Med. Sci. Law.

[43] Connell C.M., Bergeron N., Katz K.H., Saunders L., Tebes J.K. (2007). Re-referral to child protective services: The influence of child, family, and case characteristics on risk status. Child Abuse Negl..

[44] Usher C.L., Locklin E., Wildfire J.B., Harris C.C. (2001). Child Welfare Performance Ratings: One State’s Approach. Adm. Soc. Work.

[45] Kum H.-C., Joy Stewart C., Rose R.A., Duncan D.F. (2015). Using big data for evidence based governance in child welfare. Child. Youth Serv. Rev..

[46] Drahota A., Meza R.D., Brikho B., Naaf M., Estabillo J.A., Gomez E.D., Vejnoska S.F., Dufek S., Stahmer A.C., Aarons G.A. (2016). Community-academic partnerships: A systematic review of the state of the literature and recommendations for future research: Systematic review of community-academic partnerships. Milbank Q..

[47] Cashman S.B., Adeky S., Allen A.J., Corburn J., Israel B.A., Montaño J., Rafelito A., Rhodes S.D., Swanston S., Wallerstein N. (2008). The power and the promise: Working with communities to analyze data, interpret findings, and get to outcomes. Am. J. Public Health.

[48] Nind M. (2011). Participatory data analysis: A step too far?. Qual. Res..

[49] Currie M., King G., Rosenbaum P., Law M., Kertoy M., Specht J. (2005). A model of impacts of research partnerships in health and social services. Eval. Program Plann..

[50] Hacker K., Tendulkar S.A., Rideout C., Bhuiya N., Trinh-Shevrin C., Savage C.P., Grullon M., Strelnick H., Leung C., DiGirolamo A. (2012). Community capacity building and sustainability: Outcomes of community-based participatory research. Prog. Community Health Partnersh. Res. Educ. Action.

[51] Antwi-Boasiako K., King B., Black B., Fallon B., Trocmé N., Goodman D. (2016). Ethno-Racial Categories and Child Welfare Decisions: Exploring the Relationship with Poverty.

[52] Fallon B., Black T., Van Wert M., King B., Filippelli J., Lee B., Moody B. (2016). Child Maltreatment-Related Service Decisions by Ethno-Racial Categories in Ontario in 2013.

[53] Chabot M., Fallon B., Tonmyr L., MacLaurin B., Fluke J., Blackstock C. (2013). Exploring alternate specifications to explain agency-level effects in placement decisions regarding aboriginal children: Further analysis of the Canadian Incidence Study of Reported Child Abuse and Neglect Part B. Child Abuse Negl..

[54] Fallon B., Chabot M., Fluke J., Blackstock C., MacLaurin B., Tonmyr L. (2013). Placement decisions and disparities among Aboriginal children: Further analysis of the Canadian incidence study of reported child abuse and neglect part A: Comparisons of the 1998 and 2003 surveys. Child Abuse Negl..

[55] Fluke J.D., Chabot M., Fallon B., MacLaurin B., Blackstock C. (2010). Placement decisions and disparities among aboriginal groups: An application of the decision making ecology through multi-level analysis. Child Abuse Negl..

[56] Sinha V., Trocmé N., Fallon B., MacLaurin B. (2013). Understanding the investigation-stage overrepresentation of First Nations children in the child welfare system: An analysis of the First Nations component of the Canadian Incidence Study of Reported Child Abuse and Neglect 2008. Child Abuse Negl..

[57] Trocmé N., Knoke D., Blackstock C. (2004). Pathways to the overrepresentation of aboriginal children in Canada’s child welfare system. Soc. Serv. Rev..

[58] First Nations Centre (2007). OCAP: Ownership, Control, Access and Possession. Sanctioned by the First Nations Information Governance Committee, Assembly of First Nations.

[59] Canadian Institutes of Health Research, Natural Sciences and Engineering Research Council of Canada, Social Sciences and Humanities Research Council of Canada (2014). Tri-Council Policy Statement: Ethical Conduct for Research Involving Humans.

[60] Trocmé N., Kyte A., Sinha V., Fallon B. (2014). Urgent protection versus chronic need: Clarifying the dual mandate of child welfare services across Canada. Soc. Sci..

[61] Fallon B., Trocme N., Black T., Ekins A., O’Connor C., Betito P.A. (2017). Recurrence Rates by Urgent Protection and Chronic Need.

[62] Wulczyn F., Alpert L., Orlebeke B., Haight J.M. (2014). Principles, Languages, and Shared Meaning: Toward A Common Understanding of CQI in Child Welfare.

[63] Ahn H., Carter L.M., Reiman S., Hartzel S. (2017). Development of a Quality Assurance and Continuous Quality Improvement (CQI) Model in Public Child Welfare Systems. J. Public Child Welf..

[64] Ministry of Children and Youth Services (2005). Child Welfare Transformation 2005: A Strategic Plan for A Flexible, Sustainable and Outcome-Oriented Service Delivery Model.

[65] Fallon B., Trocmé N., MacLaurin B., Sinha V., Hélie S. (2015). Provincial comparisons in the Canadian Incidence Study of Reported Child Abuse and Neglect-2008: Context for variation in findings. Int. J. Child Yoth Resil..

